# Fecal Microbiota and Diet of Children with Chronic Constipation

**DOI:** 10.1155/2016/6787269

**Published:** 2016-06-23

**Authors:** Joyce Gomes de Moraes, Maria Eugênia Farias de Almeida Motta, Monique Ferraz de Sá Beltrão, Taciana Lima Salviano, Giselia Alves Pontes da Silva

**Affiliations:** ^1^Federal University of Pernambuco, Avenida Professor Moraes Rego 1235, University City, 50670-901 Recife, PE, Brazil; ^2^Mother-Child Department, Federal University of Pernambuco, Avenida Professor Moraes Rego 1235, University City, 50670-901 Recife, PE, Brazil; ^3^Department of Biotechnology, Laboratory of Immunopathology Keizo Asami (LIKA), Avenida Professor Moraes Rego 1235, University City, 50670-901 Recife, PE, Brazil

## Abstract

Many factors explain dysbiosis in chronic constipation (CC), such as a low-fiber diet. The objective of this study was to compare the fecal microbiota of constipated and nonconstipated children and their intake frequencies of food.* Methods*. This observational study included 79 children (M/F 43/36) aged six to 36 months divided into two groups: cases (39 constipated children) and controls (40 nonconstipated children). We used a structured form to collect demographic variables, conducted anthropometric assessment, and collected food intake frequency data. The fecal microbiota of the stool samples was analyzed by real-time polymerase chain reaction (PCR) using the fluorophore SYBR® Green.* Results*. Constipated children had a smaller concentration of* Lactobacillus* per milligram of stool (*p* = 0.015) than nonconstipated children, but the concentration of* Bifidobacterium* per milligram of stool (*p* = 0.323) and the intake of fruits, vegetables (*p* = 0.563), and junk food (*p* = 0.093) of the two groups did not differ. Constipated children consumed more dairy products (0.45 ± 0.8; *p* > 0.001), were more frequently delivered via caesarean section (69.2%), were weaned earlier (median: 120; 60Q_1_–240Q_3_), and had a family history of constipation (71.8%).* Conclusions*. Children with CC have a smaller concentration of* Lactobacillus* in their stools and consume more dairy products.

## 1. Introduction

Chronic constipation (CC) is frequent in children and corresponds to approximately 25% of visits to pediatric gastroenterology offices [1]. Most CC cases in small children can be explained by functional changes [2] secondary to a low-fiber diet [3] and early weaning [4].

Studies suggest that chronically constipated children have different intestinal microbiota [5, 6] and that factors such as caesarean birth, early weaning, and low-fiber diets hinder intestinal colonization by* healthy* microorganisms [7, 8].

Diet is critical among factors capable of modulating the intestinal microbiota [9–11] because saccharolytic bacteria, such as those from the genera* Bifidobacterium* and* Lactobacillus*, ferment fibers into short-chain fatty acids (SCFA), reducing intestinal pH and consequently increasing intestinal motility [12–15].

It is possible that there is altered intestinal microbiota in patients with Functional Gastrointestinal Diseases repercussions on intestinal motility [16–18]. Based on this hypothesis, researchers suggest a beneficial effect of the use of probiotics in the management of constipation, especially* Lactobacillus* and* Bifidobacterium* [18–20] what motivated us to conduct this study and we decided to compare the amount of bacteria from the genera* Lactobacillus* and* Bifidobacterium* in the fecal microbiota of constipated and nonconstipated children using SYBR Green analysis and investigate the children's intake of fruits, vegetables, junk food, and dairy products.

## 2. Methods

This observational study was conducted from March to December 2013, in a primary care unit, with 79 children from low-income families. The children were divided into two groups, recruited in consecutive order, and diagnosed according to the Rome III Diagnostic Criteria [21]: cases, 39 children with CC aged six months to less than three years, and controls, 40 children without CC matched for age.

The cases were children who had at least two of the following parameters for at least one month: two or fewer defecations a week; at least one episode of incontinence a week; history of excessive stool retention; at least one episode of hard or painful bowel movement a week; and history of large diameter stools that may obstruct the toilet [22]. The controls did not have a history of constipation. The control group consisted of healthy children assisted in the primary care unit, who had no previous history of constipation and had none of the parameters used in Roma III Criteria.

All children with a diagnosis of genetic syndromes, delayed neuropsychomotor development, chronic encephalopathy, celiac disease, or Hirschsprung's disease; evidence of constipation secondary to anatomic (anal stenosis) or functional (intestinal pseudo-obstruction) abnormalities; and evidence of constipation secondary to cow milk allergy were excluded. The mothers were informed orally of the study objectives, operationalization, and ethical aspects and signed an informed consent form before their children were enrolled in the study.

This study was approved by the Research Ethics Committee of the Federal University of Pernambuco under protocol number 165,098, on December 05, 2012, and followed all the rules established by Resolution 196 of the National Council of Health.

The structured form collected the following data: sex, age, history of prematurity, family history of constipation, time of weaning, and nutritional status. A food frequency questionnaire (FFQ) with 96 foods collected the children's dietary data.

The children's nutritional status was classified according to their body mass index- (BMI-) for-age *z*-scores using the curve provided by the World Health Organization (WHO) for children aged 0 to 5 years. The following cut-off points were used: extreme underweight when *z*-score < −3; underweight when −3 ≤ *z*-score < −2; normal weight when −2 ≤ *z*-score ≤ +1; risk of overweight when +1 < *z*-score ≤ +2; overweight when +2 < *z*-score ≤ +3; and obesity when *z*-score > +3 [27]. BMI-for-age was calculated by the software WHO* Anthro Plus* of WHO.

After nutritional status assessment, a stool sample was collected from each child and placed in a sterile polypropylene container. The stool samples were transported in ice-filled coolers and stored in a freezer at −18°C until molecular analysis.

The FFQ was assessed as recommended by de Fornés et al. [28] with adaptations, where the general intake frequency is converted into scores. Six food groups were constituted: grains, tubers, and roots (group I); beans and high-protein plant foods (group II); fruits and vegetables (group III); milk and dairy products (group IV); meats and eggs (group V); and junk food (group VI).

The answers (never, daily, weekly, monthly, and annually) were transformed into monthly intake frequencies. Foods were consumed daily and never received maximum (*S* = 1) and minimum (*S* = 0) scores, respectively. Intermediate scores were given by the formula *S* = *n*/30, where *n* is the number of days in a month that the child consumed a given food [28, 29].

The Laboratory of Immunopathology Keizo Asami of the Federal University of Pernambuco (LIKA/UFPE) analyzed the stool samples by real-time polymerase chain reaction (PCR) using the fluorophore SYBR Green. The commercial kit QIAamp DNA Stool*™* (QIAGEN, Venlo, Netherlands) was used for extracting DNA from the samples as instructed by the manufacturer. The amplification reactions used reagents standardized for real-time PCR (Universal PCR Master Mix) and a set of primers specific for the genera* Lactobacillus* and* Bifidobacterium* and total bacteria. The primers were acquired from studies on fecal microbiota analysis by real-time PCR (SYBR Green) (Table 1).

The statistical analyses were performed by the software Statistical Package for Social Sciences (SPSS) for Windows version 13.0 (SPSS Inc., Chicago, IL, USA). The numeric variables were represented by measures of central tendency and dispersion. The chi-square test and Fisher's exact test measured associations between the categorical variables. The Kolmogorov-Smirnov test determined whether the quantitative variables had a normal distribution. Student's *t*-test and the Mann-Whitney test compared the variables with and without normal distribution, respectively, especially variables related to the microbiota. Statistical analysis of microbiota-related data was performed by the software* Graphpad Prism* using the same criteria cited above. Since food intake frequencies are ordinal measurements, they were expressed as medians and interquartile ranges. The significance level was set at 5% (*p* ≤ 0.05).

## 3. Results

A total of 79 children participated in the study, 39 cases and 40 controls. Thirty case and 29 control stools were analyzed. Total bacteria could not be amplified in one case. The other study variables of the children did not differ regardless of fecal microbiota analysis.

Table 2 shows the general characteristics of the sample.

Most constipated children had a positive family history of constipation (71.8%; *p* = 0.016), a higher frequency of cesarean delivery (69.2%; *p* = 0.084), and a shorter duration of breastfeeding (median: 120; 60Q_1_–240Q_3_; *p* = 0.006).

The groups had similar intakes of fruits and vegetables (*p* = 0.563). However, constipated children consumed more dairy products (*p* < 0.001) and junk food (*p* = 0.093) (Table 3), combined with a significantly smaller amount of* Lactobacillus* genus per milligram of stools (*p* = 0.022) when compared to children not constipated (*p* = 0.015) (Figure 3).

**Figure 1:**
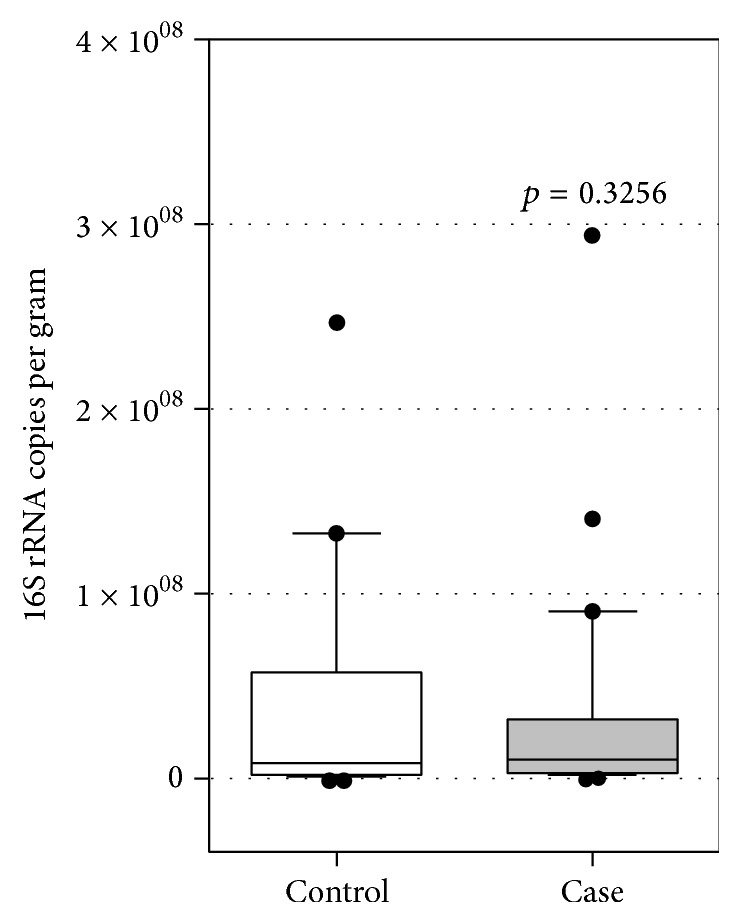
Total bacterial 16S rRNA gene copies detected per gram of healthy (control) and constipated (case) children stools (*n* = 29 for each population). Box plots show the 10th to 90th percentile range of the data within the box, with outliers indicated as dots. *p* value with 95% CI was calculated by *t*-test.

**Figure 2:**
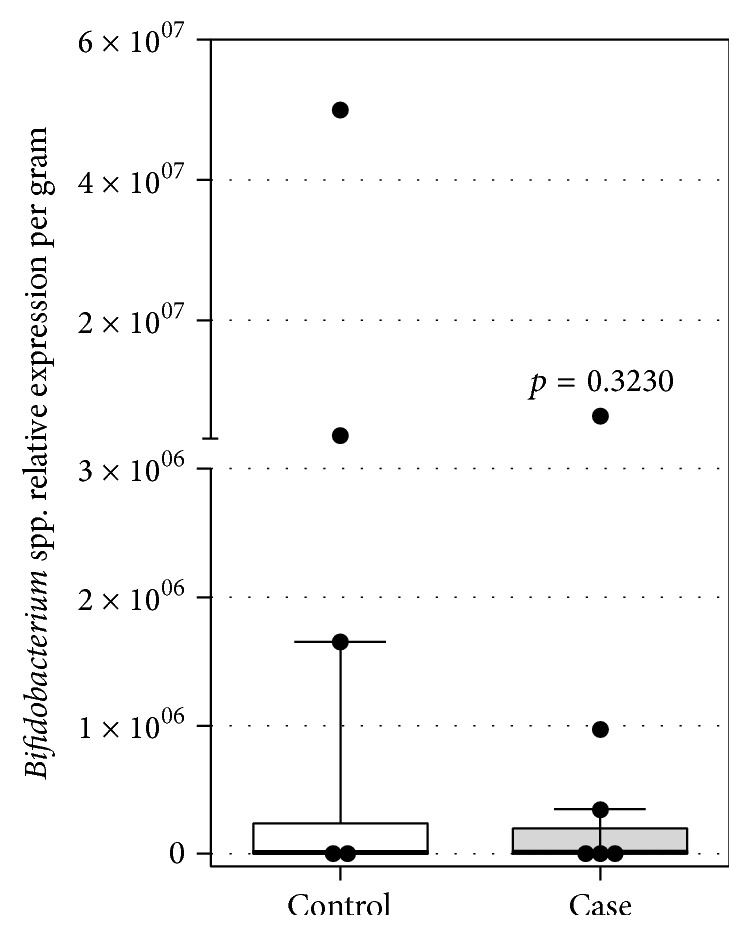
Relative expression of* Bifidobacterium* spp. analyzed by RT-qPCR in stools of healthy (control) and constipated (case) children (*n* = 29 for each population) (*Bifidobacterium* genus per mg of stool of the two study groups).

**Figure 3:**
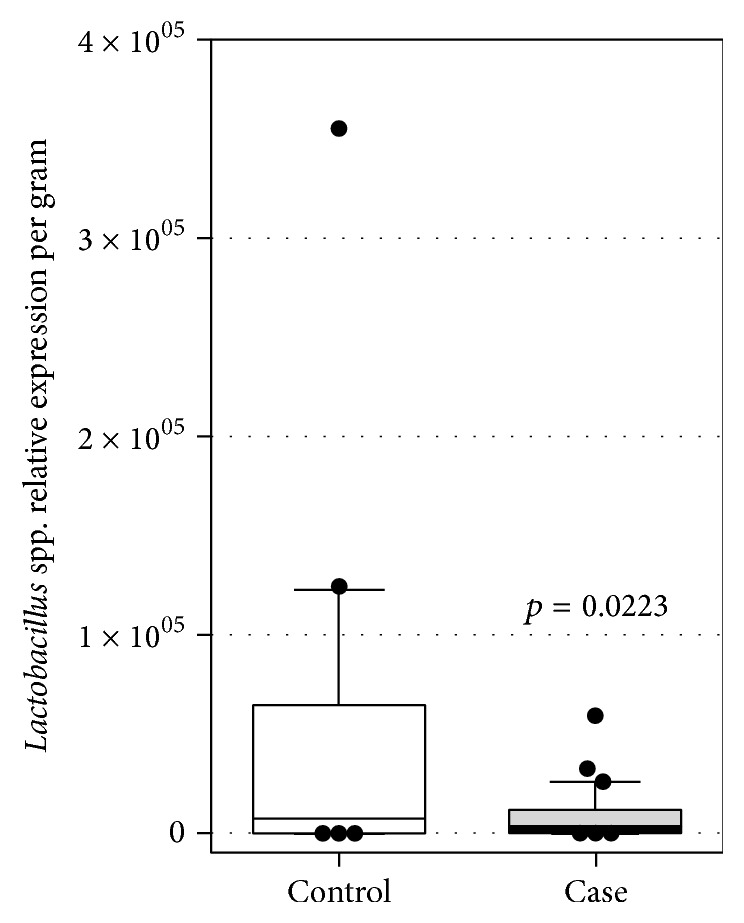
Relative expression of* Lactobacillus* spp. analyzed by RT-qPCR in stools of healthy (control) and constipated (case) children (*n* = 29-30 for each population).

The two groups had similar total bacteria counts per milligram of stool (*p* = 0.325) (Figure 1) and similar counts of the genus* Bifidobacterium* per milligram of stool (*p* = 0.323) (Figure 2).

## 4. Discussion

Constipated children had a smaller number of* Lactobacillus* per milligram of feces and the same number of* Bifidobacterium* as nonconstipated children, which may be characterized as dysbiosis [30]. Conceptually, both qualitative and quantitative changes in intestinal microbiota as bacterial overgrowth are classified as dysbiosis [31]. Constipated children consumed dairy products and sweets more frequently.

Diet modulates the intestinal microbiota significantly [11, 32, 33]. Some nutrients in excess, such as simple sugars, proteins [34, 35], and some types of lipids, are capable of reducing bacterial genera that promote intestinal motility [10, 35]. On the other hand, it is described that some fibers improve intestinal motility by stimulating bacterial genera with fermentative and saccharolytic activities [11, 15, 35–37].

High intakes of simple sugars, fatty acids, and proteins change the composition of the intestinal microbiota [9, 38, 39] and have been associated with intestinal constipation in children. Crowley et al. found that cow milk protein was positively associated with intestinal constipation in children aged three to twelve years, but the mechanisms had not been fully elucidated [40]. Kocaay et al. verified a positive association between intestinal constipation and cow milk intake in excess of 250 mL per day, which the authors believed was caused by the saponification of fatty acids with calcium [41]. Other authors consider that excess protein intake changes the intestinal microbiota [9, 38, 39].

It is noteworthy that constipated children consumed sweets more frequently and dairy products significantly more frequently. It is possible that the high intake frequency of these foods by constipated children led them to consume fewer foods with higher fiber content, such as fruits and vegetables, for example.

On the other hand, foods with high simple sugar, fatty acid, and protein contents, such as dairy products and sweets, are associated with fewer* Lactobacillus* [9, 10] genera in children's intestinal microbiota because they promote the growth of other bacterial genera [10]. Hence, these mechanisms may justify the low* Lactobacillus* count per milligram of feces in samples of children with high intake of sweets and dairy products.

In agreement with the study results, other observational studies did not detect a smaller number of bacteria from the genera* Bifidobacterium* in the fecal microbiota of constipated children and children with low fiber intake [6, 42]. Using the table of the Association of Official Analytical Chemists to determine dietary fiber intake, Aguirre et al. did not find a significant difference in the fiber intake of constipated and nonconstipated children [42].

However, studies that related fiber intake to intestinal constipation are controversial, possibly because of the different methods and tables used for determining fiber intake [43, 44]. Based on the fiber intake recommended by the Academy of Pediatrics, Ip et al. found that constipated children consume less fiber than nonconstipated children [45]. The methods used by Aguirre et al. and Ip et al. to calculate fiber intake differed from the method used by the present study [42, 45].

De Filippo et al. reported that children with high-fiber diets have more bifidobacteria in their intestinal microbiota than children with low-fiber diets [11]. Bernal et al. obtained similar results [46].

Therefore, fruit and vegetable intake seems to increase the number of* Bifidobacterium* in fecal microbiota [47], and children who consume fewer fruits and vegetables have more constipation than children who consume adequate amounts of these foods [48–50]. The number of* Bifidobacterium* by milligram of feces was not associated with fruit and vegetable intake frequency in either study group.

Conceptually, the role played by fibers on intestinal motility is associated with its action on the intestinal microbiota. People are incapable of hydrolyzing this nutrient, so the intestinal microbiota synthesizes hydrolases that break down fibers. As the intestinal microbiota ferments fiber, it produces short-chain fatty acids (SCFA), which reduce intestinal pH. Low intestinal pH reduces intestinal transit time [51, 52] and stimulates smooth muscles, actions that promote peristalsis. Low intestinal pH also promotes the growth of beneficial intestinal microbiota [10, 53], especially bacteria from the genera* Bifidobacterium* [10, 15] and* Lactobacillus* [13, 14], and inhibits the growth of pathogens [10, 35, 37, 54].

Bacteria from the genera* Bifidobacterium* and* Lactobacillus* are important constituents of children's microbiota [55, 56]. Constipation during childhood promotes qualitative and quantitative changes in the intestinal microbiota, especially with respect to these genera [56–58], which influences peristalsis. However, the exact mechanisms have not yet been fully elucidated [59–61].

Most study children with constipation had a family history of constipation along with a higher frequency of caesarian delivery and shorter breastfeeding duration. This fact may be justified by shared food habits and intestinal microbiota between family members [62]. This frequency is high and above the frequency found in other European studies, which found frequencies ranging from 13% to roughly 54% [41, 45, 63–65].

However the study frequency was very close to that found by a Brazilian study, which attributed the high frequency of constipation among family members (81.6%) to their common food habits [62]. This difference between studies may stem from the method used, since family members with constipation were presently identified by ROMA III while other studies used a questionnaire or retrospective assessment of the medical records.

Vaginal delivery and breastfeeding are thought to be key players in the colonization and succession of healthy microorganisms in children's digestive tract [41, 42, 66–68]. In agreement with our findings, Mitsou et al. found that changes in children's intestinal microbiota are associated with a higher frequency of caesarian delivery and characterized by a smaller number of* Lactobacillus* in their intestinal microbiota [67].

Breast milk contains not only* Lactobacillus* in its composition [69–71] but also prebiotics that favor their proliferation in children's intestines [72–74]. The mother's intestinal microbiota influences the microbiota present in breast milk. Breast milk bacteria differ from skin bacteria. During pregnancy, high intestinal permeability allows the translocation of intestinal bacteria to Peyer's patches and lamina propria, and mononuclear cells then transfer them to the mammary glands [75, 76].

During vaginal delivery, the mother's microbiota is transferred to the child by a process called verticalization. In other words, the child is inoculated orally by the mother's vaginal and intestinal microbiota as the child passes through the birth canal. Thus, children born by vaginal delivery have more diverse intestinal microbiota, which is very similar to the mother's intestinal microbiota. On the other hand, children born by caesarian delivery are colonized later by the bacteria present in the environment [30, 76–80].

Studies have used cultures to analyze fecal microbiota [6, 58] even though only a small percentage of the species that compose fecal microbiota can be analyzed by this technique. The present study used molecular techniques to analyze fecal microbiota.

The present results corroborate those of Zoppi et al., who used cultures to analyze the intestinal microbiota of children and also found that constipated children have a smaller number of* Lactobacillus* and the same number of* Bifidobacterium* as nonconstipated children, which has been characterized by the authors as dysbiosis [6].

Despite the scarcity of studies on the fecal microbiota of constipated individuals, there is a reasonable number of studies on the use of probiotics to revert this condition [18–20, 81–85]. Intervention studies have shown that the use of probiotics in constipated children improves the symptoms [20, 85], suggesting that their use is capable of modulating the fecal microbiota, which is associated with an improvement in childhood constipation [86, 87]. It may be indirectly inferred that changes in the intestinal microbiota are a major factor in the pathophysiology of constipation in children.

## 5. Conclusion

Thus, our results showed that constipated children had a positive maternal history for constipation associated with a higher rate of cesarean delivery, a shorter time of breastfeeding, and increased consumption of junk foods and dairy products combined with dysbiosis. No empirical basis for inferring the dysbiosis is causative factor or a result of constipation, which in its complex and multifactorial character needs to have these and more other factors involved most be studied.

## Figures and Tables

**Table 1:** *Primers *of groups in real-time polymerase chain reaction.

Group or species	Oligonucleotide sequence (5′-3′)	Verification media	Reference
Eubacteria (total bacteria)	UniF340: ACTCCTACGGGAGGCAGCAGT	11 strains of Eubacteria	[23, 24]
	UniR514: ATTACCGCGGCTGCTGGC		

*Lactobacillus* spp.	gLactoF: TGGAAACAGRTGCTAATACCG	21 strains of *Lactobacillus*	[25]
	gLactoR: GTCCATTGTGGAAGATTCCC		
	LacAcR: GCGGAAACCTCCCAACA		

*Bifidobacterium* spp.	gBifidF: CTCCTGGAAACGGGTGG	11 strains of *Bifidobacterium*	[26]
	gBifidR: GGTGTTCTTCCCGATATCTACA		
	BiLonR_2: TACCCGTCGAAGCCAC		

**Table 2:** General characteristics of the sample.

Variables	Constipated children (39) *n* (%)	Nonconstipated children (40) *n* (%)	Total *n* (%)	*p* value
*Sex n (%)*				0.305^*▪*^
Female	15 (38.5)	21 (52.5)	36 (45.6)	
Male	24 (61.5)	19 (47.5)	43 (54.4)	

*Age (months)* ^◊§^	16.7 ± 8.3	15.6 ± 6.7		0.532^*▪*^

*Nutritional status n (%)*				0.597^†^
Underweight/normal	31 (79.5)	28 (71.8)	59 (75.6)	
Overweight/obese	8 (20.5)	11 (28.2)	19 (24.4)	

*Delivery n (%)*				0.084^*▪*^
Vaginal	12 (30.8)	21 (52.5)	33 (41.8)	
Caesarean	27 (69.2)	19 (47.5)	46 (58.2)	

*Premature n (%)*				1.00^†^
Yes	3 (7.7)	3 (7.5)	6 (7.6)	
No	36 (92.3)	37 (92.5)	73 (92.4)	

*Family history of constipation n (%)*				0.016^*▪*^
Yes	28 (71.8)	17 (42.5)	45 (57)	
No	11 (28.2)	23 (57.5)	34 (43)	

*Breastfeeding duration (days)* ^**∗**^	120 (60; 240)	270 (180; 360)		0.006^*▪*^

^§^Mean ± standard deviation. ^*▪*^Pearson's chi-square test. ^†^Fisher's exact test. ^◊^Student's *t*-test. ^*∗*^Median and percentiles (25th and 75th), Mann-Whitney test.

**Table 3:** Median and interquartile ranges of the food intake frequency scores of constipated and nonconstipated children.

Variables	Constipated children (39)	Nonconstipated children (40)	*p* value
Group 1 (grains, tubers, and roots)^**∗**^	0.24 (0.17; 0.30)	0.22 (0.15; 0.28)	0.220
Group 2 (beans and other high-protein plant foods)^**∗**^	0.20 (0.08; 0.20)	0.20 (0.08; 0.20)	0.858
Group 3 (fruits and vegetables)^**∗**^	0.21 (0.12; 0.31)	0.20 (0.12; 0.28)	0.563
Group 4 (milk and dairy products)^§^	0.45 ± 0.18	0.27 ± 0.17	<0.001
Group 6 (junk food)^§^	0.15 ± 0.11	0.11 ± 0.09	0.093

^*∗*^Median and percentiles (25th and 75th), Mann-Whitney test.

^§^Mean ± standard deviation.
